# High‐Throughput Inertial Focusing of Micrometer‐ and Sub‐Micrometer‐Sized Particles Separation

**DOI:** 10.1002/advs.201700153

**Published:** 2017-05-30

**Authors:** Lei Wang, David S. Dandy

**Affiliations:** ^1^ School of Biomedical Engineering Colorado State University 80523 Fort Collins CO USA; ^2^ Chemical and Biological Engineering Colorado State University 80523 Fort Collins CO USA

**Keywords:** cyanobacteria, high‐throughput, inertial focusing, micrometer and sub‐micrometer particles

## Abstract

The ability to study individual bacteria or subcellular organelles using inertial microfluidics is still nascent. This is due, in no small part, to the significant challenges associated with concentrating and separating specific sizes of micrometer and sub‐micrometer bioparticles in a microfluidic format. In this study, using a rigid polymeric microfluidic network with optimized microchannel geometry dimensions, it is demonstrated that 2 µm, and even sub‐micrometer, particles can be continuously and accurately focused to stable equilibrium positions. Suspensions have been processed at flow rates up to 1400 µL min^−1^ in an ultrashort 4 mm working channel length. A wide range of suspension concentrations—from 0.01 to 1 v/v%—have been systematically investigated, with yields greater than 97%, demonstrating the potential of this technology for large‐scale implementation. Additionally, the ability of this chip to separate micrometer‐ and sub‐micrometer‐sized particles and to focus bioparticles (cyanobacteria) has been demonstrated. This study pushes the microfluidic inertial focusing particle range down to sub‐micrometer length scales, enabling novel routes for investigation of individual microorganisms and subcellular organelles.

## 1 Introduction

The ability to continuously and reliably concentrate and separate small diameter bioparticle (*D*_p_ ≤ 2 µm) suspensions, such as bacteria, subcellular organelles, and even virus that are flowing through microchannels, offers significant potential for biomedical,^1^ environmental,^2^, ^3^ food analysis,^4^ and biofuel production^5^ applications. Typically, bioparticle concentration and separation are accomplished through industrial or laboratory centrifugation, where there is a correlation between the size and density of the particles and the rate that they separates from a heterogeneous mixture.^6^ When the particle size is very small and its density is comparable to the mixture medium, as with bacteria, virus, and subcellular organelles, a high‐speed or even ultrahigh‐speed centrifuge operating for a long period of time is required.^6^ For moderate volumes of small bioparticle suspensions, centrifugation will work but may result in mechanical damage to the cells due to high shear forces.^7^, ^8^ In typical clinical samples where the bacteria/virus numbers are often low and the sample volume is small, centrifugation may be inadequate at concentrating or separating the suspension constituents.^9^, ^10^ On the other hand, in industry applications such as cyanobacteria harvesting for biofuel production, large‐scale concentration of a dilute cell suspension entails significant power requirements associated with high‐speed centrifugation.^11^

Consequently, a simple but robust platform able to provide significant improvements over current concentration techniques is needed. Ideally, a concentration/separation device for small diameter particle/bioparticle suspensions would be as simple as possible in its design, not require additional reagents or external electronics, and be inexpensive to fabricate and operate. Also, the device should be readily scalable so that the basic technology could be applied to sample volumes ranging from milliliters to hundreds of liters. And the device needs to be sufficiently small to potentially integrate with other point‐of‐care platforms.

The use of microfluidics has streamlined many traditional laboratory techniques, due to the advantages of ease to operation, low‐cost, and miniaturized size.^12^ In the specific application to particle/bioparticle concentration and separation, inertial focusing is a very promising approach that relies solely on channel geometry and intrinsic hydrodynamic forces.^13^, ^14^, ^15^, ^16^, ^17^, ^18^ The application of inertial focusing devices has been used for precise manipulation of erythrocyte‐sized cell suspensions for a number of applications in clinical diagnostics.^14^, ^19^, ^20^, ^21^ However, separation of smaller, micrometer‐sized bioparticles is challenging using current inertial microfluidics approaches.^16^, ^21^, ^22^, ^23^

To address the challenge, a better understanding on the mechanism of inertial focusing is necessary to find potential solutions. The inertial focusing phenomenon arises from lateral forces exerted on particles in a dilute suspension as they are transported in flow with a nonuniform velocity profile under laminar conditions. The equilibrium migration location of particles depends on a number of factors, including the ratio of the particle size to the channel dimensions and the Reynolds number, *Re*,^16^, ^20^ which is a dimensionless parameter quantifying the ratio of inertial forces to viscous forces.^14^ In laminar channel or tube flow, each particle experiences a force associated with the parabolic velocity profile. This force, the shear gradient lift force (*F*_SL_), pushes particles away from channel centerline. At the same time, the channel wall exerts a wall‐effect lift force (*F*_WL_) that pushes the particles away from the channel or tube wall.^16^ The net lift force (*F*_L_) in a rectangular channel can be expressed as^16^

(1) $$F_{L}\;=\;\frac{\rho U_{m}^{2}a^{4}}{D_{h}^{2}}f_{c}$$

where ρ is the fluid density, *U*_m_ is the maximum channel velocity, *a* is the particle diameter, *f*_c_ is the lift coefficient, and *D*_h_ = 2*hw*/(*h* + *w*) is the hydraulic diameter, where *h* and *w* are the height and width of the channel cross section, respectively. As shown in Equation (1), the net lift force will decrease significantly with relatively small changes in particle size due to the fourth‐order dependence. To overcome this effect and obtain a focused particle stream, smaller cross sections and larger velocities are needed. In this study, a channel with appropriately scaled dimensions for small particle/bioparticles suspensions has been designed and fabricated.

The working flow rates in current inertial focusing microfluidics platforms are usually at modest Reynolds numbers (*Re* ≈ 100),^16^, ^22^, ^24^ which limits the ability to focus small particles at high throughput in a single microchannel. However, when higher velocities are required, the low elastic modulus of the widely used material polydimethylsiloxane (PDMS) makes its use problematic for this microfluidics application, resulting in cross‐section deformation and a loss of focusing at higher flow rates.^21^, ^23^ Other polymers with comparably simple fabrication procedures—but much higher rigidity—are needed. A benefit of using higher flow rates is the potential for reduction in channel length, from several centimeters^16^ down to millimeters. The reduced size should also lead to reduced pressure drops and pumping requirements.^16^

To get a better separation outcome, fewer particle equilibrium positions are favorable. In a tube of circular cross section, randomly distributed particles are known to focus to an annulus located six‐tenths of the distance from the axis to the tube wall,^13^ whereas in a channel of a square cross section the particles focus to four equilibrium regions centered near each face.^16^ The number of focused particle streams can be reduced by introducing curvature to the flow path.^14^ The inertia of the fluid moving through a channel bend creates secondary swirling motion, known as Dean flow.^14^, ^15^ The resulting hydrodynamic drag enhances the lateral migration of particles across the channel. There are two major classes of curved channels: a spiral geometry^18^, ^25^, ^26^ and a serpentine channel geometry with asymmetric^14^, ^15^, ^27^ or symmetric configurations.^28^, ^29^ In this design, a serpentine channel is used to reduce the number of focused particle streams. An added benefit of this slightly increased geometric complexity is a reduced flow length required to achieve focusing relative to straight channels.^16^, ^30^ The linear layout of the asymmetric curved channel has one more advantage—its ability to be parallelized, which allows for increased throughput.

In sum, a simple, robust device for inertial focusing of micrometer‐ and sub‐micrometer‐sized particles and bioparticles for concentration and separation at high throughput has been designed, constructed, and validated. There are a broad set of potential applications for this platform, such as pathogen and subcellular organelle isolation, separation of virus from bacteria, microalgae harvesting, and monitoring heterogeneous response of bacteria in drug susceptibility testing.

## 2 Results and Discussion

### 2.1 Design of the Focusing Device

**Figure 1:**
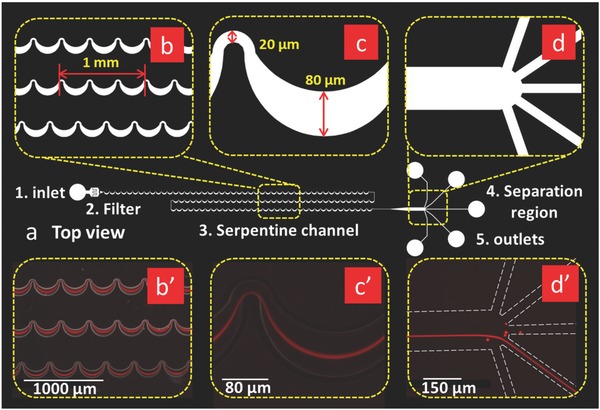
Images showing the design and use of a serpentine microfluidic network for focusing for 2 µm red fluorescent spheres. a) A schematic top view of the entire inertial focusing platform. The total length of the serpentine curved microchannel section is 4.5 cm in this design. b) Enlarged image of the serpentine curved channel. b′) Fluorescence image of the 2 µm red fluorescence particles streams in locations corresponding to (b). c) Magnified image of one serpentine unit, in which the channel width of the small bend is 20 µm and the width of the large bend is 80 µm. c′) Fluorescence images of the focused 2 µm red fluorescence particle stream at a location corresponding to (c). d) Magnified image of the isolation region. d′) Fluorescence images of the focused 2 µm red fluorescence particle stream in the isolation region. The white dashes represent the boundaries of microchannels.

The experiments done here have successfully demonstrated inertial focusing of 2 µm red and 920 nm green fluorescent polystyrene spheres using an asymmetric serpentine channel. **Figure** **1**a, at the center of Figure 1, shows the top‐view schematic of the entire microfluidic device, for which there are five functional components: (1) an inlet to introduce the homogeneous suspension; (2) a filter region to prevent channel clogging by trapping larger particles; (3) a 33.3 mm long asymmetric serpentine channel to focus the particles (doubling back to reduce the device length); (4) a separation region to isolate particle streams from media; and (5) three collection outlets. The serpentine microchannel geometry was selected because it enhances the rate of lateral particle migration. That is, at a sufficiently large value of the Dean number (*De*), at which point the Dean drag the same order of magnitude as the lift force, curved channels result in faster focusing to predicted equilibrium positions than straight channels for the same Reynolds number.^16^, ^30^ It is noted that the lift force is the dominant focusing mechanism moving particles to their equilibrium positions; the additional force from Dean flow does not focus particles, but it serves to reduce the number of equilibrium positions from four to one and to speed up the focusing process. Due to the small size of the particles, a correspondingly small channel cross section is needed to enable and maintain inertial focusing. In studies with 2 µm particles and bioparticles, the microchannel height is a uniform 10 µm and the serpentine channels have a width of 20 µm on the small curvature bends, both of which are critical parameters that determine focusing performance. One small and one large turn are defined as a unit, such that the length of three units is 1 mm. The separation channels are designed to range from 12 units (4 mm) to 100 units (33.3 mm). Figure 1b′–d′ shows the progression of inertial focusing processes for 2 µm red fluorescent polystyrene spheres in a 12 unit configuration. The particle suspension is introduced into the inlet by a syringe pump, and the suspension enters the serpentine channel region after passing through the filter. At the beginning of the suspension section (Figure 1b′), the band of red fluorescent particles spans the width of the microchannel. However, as the suspension passes through the units, the width of the particle stream continuously narrows to the point where the particles are focused (Figure 1c′). After the focused stream leaves the serpentine section it is isolated from the particle‐free liquid in the separation region.

As discussed above, the small size of the particles requires a higher flow rate (*Re*) relative to larger particles, to obtain a sufficiently large lift force. And because of the linear relationship between flow rate and pressure drop per unit length in Poiseuille flow, significant pressure is applied to the liquid. Under these circumstances, despite its fabrication simplicity and widespread use, PDMS is a not an appropriate choice for the microchannel material; pressure‐induced deformation of the channel cross section results in a total loss of the focusing effect.^21^, ^23^ Thermoset polyester (TPE) was identified as an alternative polymeric material with similar fabrication procedures to PDMS as well as optical transparency, but with much higher rigidity.^21^ Young's modulus for TPE is ≈1.2 GPa, or 1000× higher than 1:10 PDMS.^21^ The inertial focusing microfluidic chip was fabricated in TPE by single‐layer soft photolithography with several processing changes from reported methods^31^, ^32^, ^33^, ^34^, ^35^ to obtain needed versatility for a wider range of applications. First, a 3 min UV light curing step^31^, ^33^, ^35^ was replaced with a 10 min 65 °C thermal cure. This modification eliminates the need for photoinitiator in the resin mix for UV curing, and results in improved optical transparency of the TPE layer and also reduces the cost for chip fabrication. Second, although fully cured TPE is a rigid polymer, it was still possible to use standard PDMS punches to create inlet and outlet ports by doing a partial thermal cure, punching the inlets and outlets, and then completing the cure with room‐temperature O_2_ plasma treatment for sealing. The detailed procedure is illustrated in Figure S1 (Supporting Information) and described in the Experimental Section.

### 2.2 Effect of Particle Size

Within the curved channel region, the theory associated with the superposition of the lateral lift forces and secondary flow is complicated, but there is a dimensionless parameter, the inertial force ratio,^16^ that quantifies the magnitude of this effect

(2) $$R_{f}\;=\;\frac{a^{2}R}{h^{3}}$$

**Figure 2:**
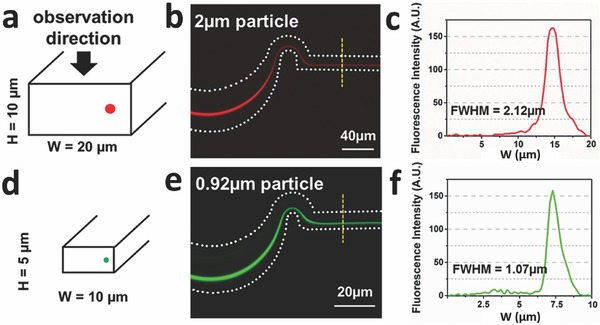
The effect of particle size on focusing efficiency. a) The channel cross section of 20 µm × 10 µm is designed for 2 µm particle focusing. b) Fluorescence image of 2 µm red fluorescent particles in the final curve of the serpentine microchannel. A single equilibrium focusing location is seen in the straight channel region. c) The corresponding fluorescence intensity profile across the width (*w*) of the straight channel region. d) The channel cross section of 10 µm × 5 µm is designed for 0.92 µm particle focusing. e) Fluorescence image of 0.92 µm green fluorescent particles in the final curve of the same serpentine microchannel. f) The corresponding fluorescence intensity profile across the width (*w*) of the straight channel region. The FWHM is calculated from a fitted Gaussian curve. The white dashed lines represent the microchannel boundaries.

This parameter contains the particle size *a*, the largest radius of curvature, *R*, and the smallest channel dimension, which is *h* in this device. When *R*_f_ > 0.04, the coupling of lift force and Dean flow will guide particles to a stable equilibrium position.^16^ By design, for the 2 µm particles in the network shown in Figure 1 the ratio is *R*_f_ = 0.6, which is well above the necessary threshold. **Figure** **2**b shows results for focusing experiments with a 0.01 v/v% suspension of the 2 µm spheres at a flow rate of 500 µL min^−1^ (*Re* = 554). The streak width, that is, the full width at half maximum (FWHM) of the intensity profile is 2.12 µm at this flow rate. When the measured FWHM is less than twice the particle diameter, the particle stream is defined as focused.^16^ There is a common view that it is difficult to precisely focus microparticles in asymmetric serpentine channels^36^ because the steak widths are often two to three times the particle diameter in size.^27^, ^37^ However, the results from this study show that the streak width is essentially the same as the particle diameter at low particle concentrations, strongly indicating that the suspension flows single file along a pathline. The ability to truly focus particles in a curved channel network depends strongly on two quantities, the flow rate and channel cross‐section dimensions. As indicated above, the Reynolds number in this experiment significantly exceeds 100, which is a typical value reported in other inertial focusing studies.^27^, ^37^ This operating condition is clearly important since the magnitude of the lift force scales as the square of the maximum fluid velocity, as shown in Equation (1). At the same time, the magnitude of the secondary flow velocity, *U*_D_, also varies as the square of that maximum velocity as

(3) $$U_{D}\;\approx \;\frac{De^{2}\mu }{\rho h}$$

where *D*_e_ = *Re*(*h*/2*R*)^1/2^ is the Dean number, and *µ* and ρ are the liquid dynamic viscosity and density, respectively.^18^ For these studies the cross‐section dimensions and particle size are fixed, so flow rate plays a major role in the focusing process. The reason that the focusing effect is lost under these conditions when using PDMS is also evident in Equation (1); the higher pressure expands the channel cross section like a balloon, resulting in unwanted increases in *D*_h_.

A suspension of smaller 0.92 µm spheres was also flowed through this serpentine network at the same flow rate and concentration as the 2 µm spheres. As shown in Figure S2 (Supporting Information), the FWHM streak width is 4.10 µm, which is 4.5 times the 0.92 µm sphere diameter. This relatively reduced focusing effect is simply due to the smaller particle size and resulting net lift force. However, despite the fact that these particles are, technically, not focused, the lift ratio is *R*_f_ = 0.13 and the relative narrowness of the streak width is significant because the magnitude of the lift force on the 0.92 µm spheres is less than 5% of that experienced by 2 µm spheres. The inertial migration effect is not sufficiently large enough to more tightly group the smaller particles, but it nevertheless displaces them with surprising efficiency. These results indicate that further tuning of channel dimensions offers the possibility of focusing sub‐micrometer particles. In follow on studies, a microchannel with reduced cross section (*w* = 10 µm and *h* = 5 µm) was designed and tested with the 0.92 µm green fluorescent microspheres at the same flow rate. There, the FWHM is 1.07 µm, as shown in Figure 2f. This result demonstrates that sub‐micrometer‐sized particles can also be focused using inertial forces. One disadvantage of this separation technique is that the fixed channel dimensions constrain operation to a relatively narrow range of particle sizes. However, it is possible to design a serial microchannel network configuration with different channel cross‐section dimensions in each stage or segment, whereby particles are focused and removed in order of decreasing particle size. For similar‐sized particles (e.g., 2 and 3 µm), a single channel could be used to produce a separation of the two sizes. Both sizes would be focused, but on distinguishable pathlines, and the outlet microchannel configuration designed based on knowledge of the different equilibrium positions.

### 2.3 Effect of Channel Length

**Figure 3:**
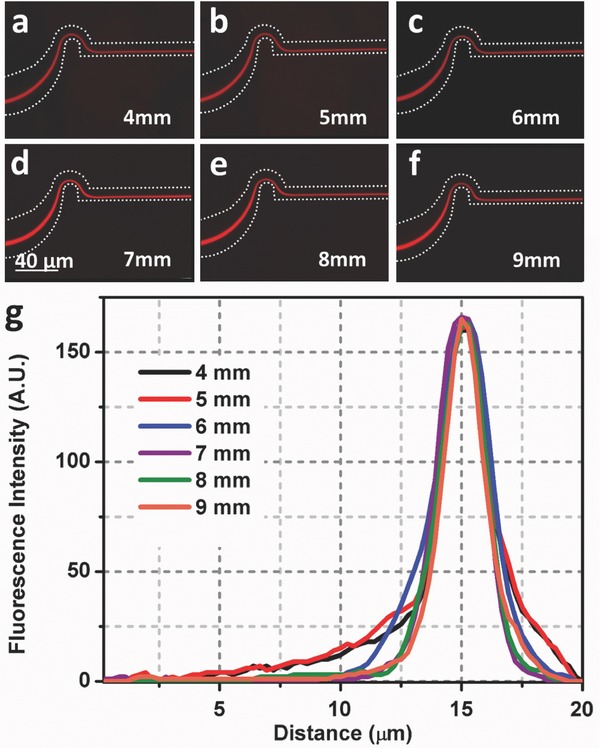
The dependence of focusing efficiency on serpentine focusing region length. a–f) Fluorescence images of 2 µm red fluorescent particles in the final curve of serpentine microchannel for channel lengths ranging from 4 to 9 mm. g) The fluorescence intensity profiles across the width of the straight channel for each of the serpentine channel lengths. The white dashes represent the microchannel boundaries.

The channel length is ultimately a critical factor in fabrication cost and power consumption, as well as the ability to scale up to process larger volumes, for example, in the mL s^−1^ range. A logical issue to address, then, is the minimum length of the serpentine section required to focus the micrometer‐sized particles. Typical channel lengths reported in the literature are on the order of several centimeters.^14^, ^17^, ^36^, ^37^ In this study, 100 units of the serpentine channel (33.3 mm total length) were chosen for focusing the 2 µm spherical particles in large part because of much lower flow rates that were initially investigated. As shown in **Figure** **3**, this length did result in very good focusing characteristics. Subsequently, serpentine channel lengths ranging from 4 to 9 mm have been designed and tested. The flow rate is fixed at 700 µL min^−1^ (*Re* = 776) in all runs, and the 2 µm fluorescent spheres introduced at a concentration of 0.01 v/v%. The fluorescence images and scanned profiles are shown in Figure 3. For all of the different channel lengths the microspheres are tightly focused into a single particle stream and the lateral position of each particle stream is the same regardless of focusing region length. The minimum serpentine channel length considered here, 4 mm, is almost one‐tenth that of the most reported inertial focusing channel lengths.^14^, ^17^, ^22^, ^36^ Additional studies were carried out with channel lengths as small as 1 mm. Those results are summarized in Figure S3 (Supporting Information).

### 2.4 Effect of Flow Rate

**Figure 4:**
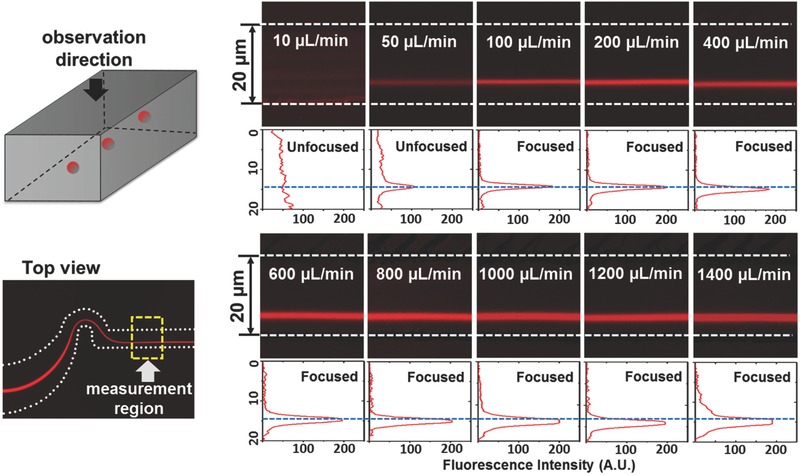
Flow rate dependence of inertial focusing efficiency for micrometer‐sized particles. The left two images show the observation direction and observation region for the images on the right. The intensity images and scans show the fluorescence intensity from 2 µm particles under the flow rates ranging from 10 (*Re* = 11.1) to 1400 µm min^−1^ (*Re* = 1550). The horizontal blue dashed line in each scan demonstrates the shift in particle streak location toward the sidewall as flow rate is increased. The white dashes represent the microchannel boundaries.

Just as reducing channel length increases throughput‐per‐footprint, so does an increase in the maximum practical flow rate. To further explore the effect of this parameter on device operation, a range of flow rates, from 10 (*Re* = 11.1) to 1400 µL min^−1^ (*Re* = 1550) were investigated. **Figure** **4** shows fluorescence images in the straight channel section immediately downstream of the serpentine region, as well as the intensity scan across the channel width. It may be seen that, for this 0.01 v/v% concentration, the degree of focusing continuously improves as flow rate is increased from 10 (*Re* = 11.1) to 100 µL min^−1^ (*Re* = 111). As the flow rate is increased beyond this value, however, there is no noticeable effect on focusing because the particles already lie on a single pathline. This result is in contrast to observations made in straight focusing channels at similar values of *Re*.^23^ Specifically, in another study using straight focusing channels, the number of focused streams varied from one to three as flow rate increased.^23^ This difference is consistent with the observation that Dean flow in serpentine channels efficiently aligns the particles into equilibrium positions that remain stable in the downstream straight channel section, and enables focusing to a smaller subset of equilibrium positions that are stable in the presence of the superposed secondary flow. As for the correlation between the strength of the secondary flow and the particle distribution, there is no straightforward relationship because a number of parameters (e.g., microchannel dimensions, particle size, channel radii of curvature, and Reynolds number) are involved, as indicated in the expressions above for the inertial force ratio and Dean number. In this design, the only adjustable parameter is Reynolds number. Therefore, the magnitude of secondary flow and Reynolds number are proportionally related by a constant. One thing that does vary with the Reynolds number or secondary flow in the current system is the lateral equilibrium position of the focused stream. Although the effect is small, the particle stream moves closer to the channel sidewall as Reynolds number or secondary flow strength is increased. Note that the apparent width of the particle streams is larger at higher flow rates. This effect is an artifact of the visualization technique that results in an increased number of particles passing through detection region during the same exposure time.

### 2.5 Effect of Suspension Concentration

**Figure 5:**
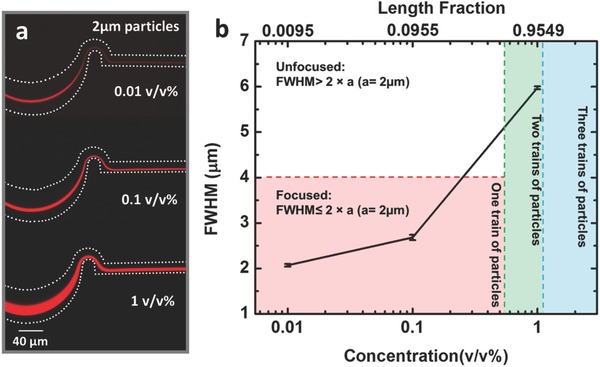
The dependence of focusing efficiency on the initial suspension concentration for 2 µm particles. a) The measured FWHM visibly increases as particle concentration is increased. b) The lower left region (pink) denotes the range of 2 µm particle concentrations that theoretically can be focused onto a single particle train (pathline). The data points in this region demonstrate that focusing is achieved. The region above this (white) represents unfocused operation. The adjacent region (green) corresponds to a range of particle concentrations (≈0.52–1.05 v/v%) that cannot physically fall onto a single pathline, but may form two adjacent particle trains. Using this as a focusing criterion, the results for the 1 v/v% suspension indicate that focusing is achieved. The final region in the plot (blue) denotes focusing for concentrations above ≈1.05 v/v%. The white dashes represent the microchannel boundaries. Error bars represent standard deviation with a sample size of three.

Another important feature of a high throughput device is the ability to focus and separate a wide range of suspension concentrations, while at the same time, recognizing that this phenomenon is concentration dependent.^16^, ^27^, ^36^ Specifically, focusing suspensions with high concentrations can be problematic due to particle–particle interactions that work against the lateral lift force mechanism. As shown in **Figure** **5**a, the width of focused 2 µm particle streams increases as particle concentration increases from 0.01 v/v% (FWHM = 2.07 ± 0.03 µm) to 1 v/v% (5.98 ± 0.03 µm). For these experiments, the flow rate was held constant at 1000 µL min^−1^ and the serpentine channel length was 4 mm. The images and plots in Figure 5 illustrate several phenomena. First, the FWHM of the particles streams are much less than twice the particle diameter when the particle concentrations are low (0.01 and 0.1 v/v%), which conclusively demonstrates focusing,^24^ as shown in the lower left (pink) region of Figure 5b. Second, the locations of the particle streams do not change appreciably as particle concentration increases. Third, it is found that, at 1 v/v%, the FWHM of the stream is 5.98 µm, which is almost three times the particle diameter. Based on the standard definition applied at the lower concentrations,^24^ this stream would be considered unfocused. However, simply due to geometric constraints there is a tendency for the particles to line up on adjacent pathlines and form “particle trains.”^38^ To explain this higher concentration phenomenon, a length fraction λ is introduced, where this quantity is the number of particle diameters per channel length.^16^ The length fraction (λ) can be related to the suspension volume fraction as

(4) $$\lambda \;=\;\frac{aA_{c}V_{f}}{V_{p}}\;=\;\frac{6whV_{f}}{\pi a^{2}}$$

where *V*_f_ is the suspension volume fraction, *A*_c_ is the channel cross‐sectional area, *w* is the channel width, and *h* is the channel height.^16^ In theory, when λ < 0.5, there can be a single train of aligned particles; the minimum number of particle trains increases from one to two when λ > 0.5, which is shown in the middle region (green) in Figure 5b, and then three trains when λ > 1, represented by the rightmost (blue) region.^38^ For the suspension concentrations used here, the corresponding λ values are 0.0095 (0.01 v/v%), 0.0955 (0.1 v/v%), and 0.9549 (1 v/v%), as shown in Figure 5b. There needs to be a minimum of two particle trains when the suspension concentration is 1%, and the focusing criterion is thereby adjusted to be FWHM ≤ 2*aN*_t_, where *N*_t_ is the minimum possible number of particle trains. In this study, then, the 1.0 v/v% suspension is focused because the FWHM < 8 µm. The upper limit of length fraction considered in this study is 1 to avoid defocusing due to strong particle interactions and crowding.^36^

As discussed above, this device is effective at focusing a dilute suspension (0.01 v/v%) of 2 µm particles at high flow rates, up to 1400 µL min^−1^, using short (4 mm) channel lengths. The ability of the device to focus and concentrate higher suspension densities—0.1 and 1.0 v/v%—was also quantified. For the outlet configurations shown in Figure S4a (Supporting Information), concentration performance (yield efficiency) was measured. As shown in Figure S4b (Supporting Information), at 500 µL min^−1^, the yield efficiencies for the higher concentrations (0.1 and 1 v/v%) rival the performance of the lowest concentration (0.01 v/v%). At 700 µL min^−1^ (Figure S4c, Supporting Information), the yield percentage is similar to the 0.01% performance, that is, above 99%. Thus, a concentration factor of over 100 with high yield efficiency is feasible if the channels were configured as a cascade, with the product stream of one flowing to the inlet of another.

A suspension concentration of 0.01 v/v% is equivalent to 10^7^ mL^−1^, and this is approximately the highest concentration of cyanobacteria in a fully cultured state (e.g., in an open pond).^39^ In other words, a 0.01 v/v% concentration covers most of the applications in industrial or laboratory settings, for example, bacteria sample preconcentration for nucleic acid extraction, or bacteria separation in water or milk. For some specific industry applications, highly concentrated bacteria suspensions are needed to extract bioproducts. Cyanobacteria‐based biofuel production is a good example for this. The suspension concentration after the culture process is not high enough in these applications for harvesting or dewatering to be economically feasible, and additional processes are needed to further increase the concentration. The higher working concentrations of 0.1 v/v% (10^8^ mL^−1^) and 1.0 v/v% (10^9^ mL^−1^) tested in this microfluidic device demonstrate that the technique is suitable for such applications.

### 2.6 Micrometer and Sub‐Micrometer Particle Separation

**Figure 6:**
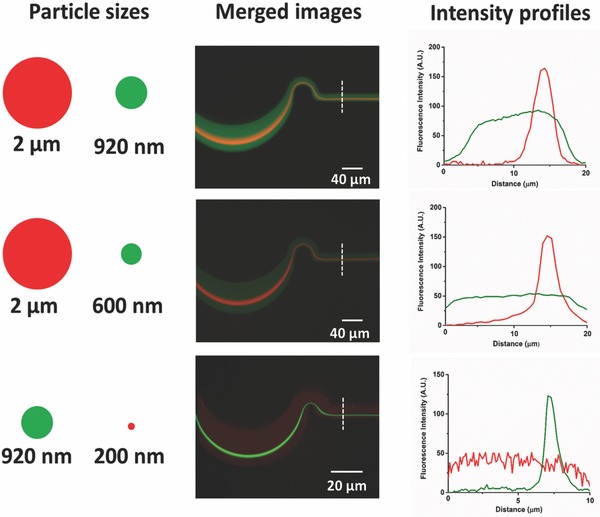
Micrometer and sub‐micrometer particle separation. The left legends show the relative particles sizes and their corresponding florescence colors; the middle images are the merged fluorescence photos of two particles with difference sizes tested in the same microchannel at the same flow rates; the right plots are the intensity profiles of corresponding particles.

There have been several recent investigations into separating micrometer and sub‐micrometer particles using microfluidic platforms.^40^, ^41^ Among these, there are few reports of inertial focusing based particle separations in the micrometer and sub‐micrometer range, but in one a spiral configuration was used to separate 3.2 and 2.1 µm particles, with 1.0 µm particles remaining unfocused.^22^ At the same time, the separation of micrometer‐ and sub‐micrometer‐sized particles/bioparticles has significant utility. For example, the separation of rod‐shaped bacterial cells (200 nm × 2–8 µm) from virus particles (20–200 nm) has immediate clinical utility as part of sample preparation or purification. In this study, separation experiments have been carried out for particles ranging from 2 µm to 200 nm. Merged fluorescence images and intensity profiles of individual particles are shown in **Figure** **6**. In the 20 µm × 10 µm serpentine microchannel, 2 µm particles with 920 or 200 nm particles were studied to demonstrate the device's ability to separate a typical bacterial cell from virus. At a modest flow rate (80 µL min^−1^), the 2 µm particles (red) can be focused while the 920 nm particles (green) remain diffuse. Although the 2 µm and 920 nm particles are relatively close in size, this work represents the first time that these two sizes have been separated using inertial focusing. The 10 µm × 5 µm serpentine channel was then used to demonstrate the extraction of 200 nm particles (0.01 v/v%) from a mixture with 920 nm particles (0.01 v/v%). The 920 nm particles (green) equilibrated near the wall of microchannel, while the 200 nm particles (red) remain unfocused and evenly distributed. This approach can be used for separation of nanoparticles and bioparticles such as bacteria and virus.

### 2.7 Application to Bioparticles

**Figure 7:**
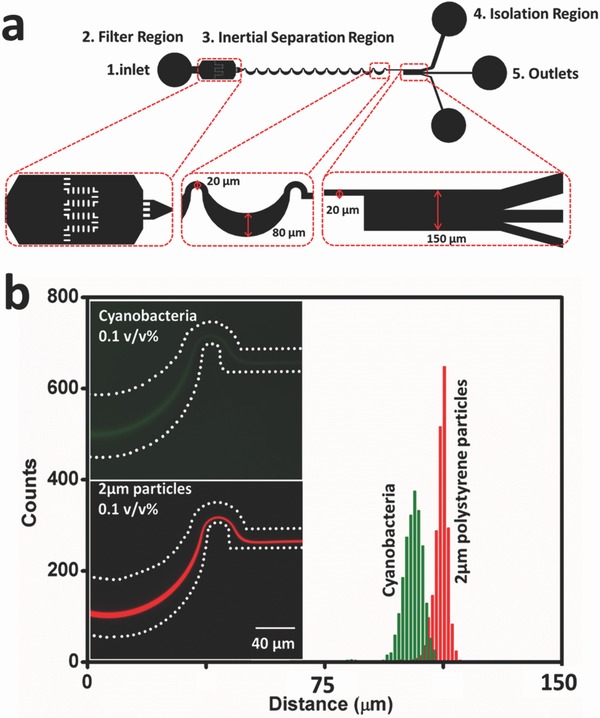
The dependence of equilibrium particle streak location on particle deformability. a) Schematics showing the layout of a serpentine microfluidic network for focusing for 2 µm spheres and cyanobateria. This figure represents a top view of the design, and enlarged images for each region. There are five functional components: an inlet for the homogeneous suspension, a filter region to prevent downstream channel clogging, an asymmetric serpentine channel to focus the particles, an isolation region to separate the particle stream from liquid media, and three collection outlets. Green dots represent cyanobacteria pathlines. b) The two fluorescence intensity images on the left part are the focused GFP‐modified cyanobacteria and red fluorescent particles in the last curve. The white dashes represent the microchannel boundaries. Shown at right are fluorescence distribution histograms of 2 µm cyanobacteria and 2 µm particles across the width of the expanded isolation region leading to the three outlet channels.

Although rigid particles have been used here as a simple model to quantify inertial focusing effects on micrometer‐size particles, bioparticles are typically not rigid and will deform under normal and shear stresses present in the flow field. To determine the effects of deformability on focusing, a suspension of cyanobacterium *Synechocystis* sp. PCC 6803, with a typical size of 2 µm^42^ was introduced into a microfluidic chip with a 4 mm long serpentine section. The cyanobacteria used here have been modified to express green fluorescent protein (GFP).^43^ **Figure** **7** shows fluorescence images of the 2 µm cyanobacteria (0.1 v/v%) and 2 µm fluorescent polystyrene spheres (0.1 v/v%) at the final curve of the focusing region. For both cases the flow rate was 300 µL min^−1^. A scan of the fluorescence intensity across the downstream isolation channel width demonstrates distinct differences in the equilibrium position of these comparably sized particles. To quantify this shift, the distributions of 5000 counts of both 2 µm red fluorescent polystyrene particles and cyanobacteria across the straight channel section are plotted. The cyanobacteria peak is 1.05 µm closer to the centerline than the polystyrene spheres. This shift might be the result of the bioparticle's shape and deformability. Although most of the cyanobacteria are spherical in shape under quiescent conditions, they will form a transient ellipsoidal shape while undergoing division, as shown in Figure S5 (Supporting Information). The equilibrium position of nonspherical particles depends on their largest dimension, and results in a shift in equilibrium position away from wall,^19^ but in this system the effect of particle shape is expected to be small. The phenomenon of a shift in equilibrium positions for rigid and deformable particles has also been observed in a study done to classify different cell types using size and deformability as distinguishing markers.^44^ The equilibrium position shift observed in this study also appears to be due to the deformability of the cyanobacteria.^17^ Our result indicates that bioparticle equilibrium position in this serpentine microchannel for micrometer‐sized particles/bioparticles depends on their shape and deformability.

## 3 Conclusion

In this study, a serpentine channel inertial focusing microfluidic system has been designed, fabricated, and tested to demonstrate that inertial migration and Dean flow can be effectively and efficiently applied to achieve focusing of dilute suspensions. The focusing of micrometer‐sized particles was accomplished through two advancements: unique chip designs for 2 and 0.92 µm particles and the use of a rigid, easy‐to‐fabricate polymer. At flow rates greater than 100 µL min^−1^, very tight focusing of 2 µm particles and cells is achieved with this new chip, and even sub‐micrometer particles are focused on the chip in a predictable manner. To increase the throughput‐per‐footprint for processing large samples, a number of factors were systematically investigated, including the effects of channel length, flow rate, and particle concentration on inertial focusing. To ensure focusing of the 2 µm particles and cells, the minimum length of the serpentine channel is found to be 4 mm, which is significantly shorter than in other published studies. In this 4 mm chip, it was observed that a stable, focused particle stream was achieved for all flow rates between 100 and 1400 µL min^−1^. A wide range of suspension concentrations—from 0.01 to 1.0 v/v%—were tested in this chip, and it was found that 0.01 and 0.1 v/v% suspensions were focused according to the standard definition. The highest concentration, 1.0 v/v%, where the particles are geometrically constrained from occupying a single pathline, suggests that a new definition for focusing be adopted that takes into account the minimum number of adjacent pathlines or trains required to accommodate all of the particles in the suspension. It was also demonstrated that the device is capable of separating disparate sized particles from one another, in particular, particles with sizes characteristic of bacteria and virus. Finally, the focusing performance of the chip for deformable bioparticles has been investigated, and it is found that bacterial cells are focused as effectively as rigid particles. This study has pushed the boundary in inertial focusing to demonstrate the ability to isolate and separate smaller, micrometer‐ and sub‐micrometer‐sized particles, which opens up new applications for bacteria and subcellular organelles in cytometry and digital microfluidics.

## 4 Experimental Section

*Microfluidic Device Fabrication*: For TPE chip fabrication, a SU‐8 2008 mold (Microchem, MA) using conventional photolithography was first created.^45^ The SU‐8 patterned silicon master was treated by vapor deposition with hexamethyldisilazane (HMDS) (Sigma‐Aldrich, MO) in a 60 °C oven for 4 h prior to replica molding with TPE. TPE was prepared by mixing 20 g resin (TAP Clear‐Lite Casting Resin, CA) with 0.2 g MEPK catalyst (TAP plastics, CA), and then the mix was stirred and degassed to remove air bubbles. A piece of PDMS was cut to form a mold surround, which confined the mix in a specific area on the master. Then the TPE mix was poured onto the mold. A piece of transparency film was used as a top cover over the mix to ensure a flat surface. The TPE is placed in a 65 °C oven for 10 min, after which the TPE replica was peeled from the master. A biopsy punch (Technical Innovations) was used to create 1.5 mm diameter inlet and outlet ports. The TPE replica and a glass substrate were then placed in a plasma chamber and pumped down to 200 mTorr, and the pieces exposed to plasma (Plasma Etch, Carson City, NV) for 1 min, at pressures between 100 and 200 mTorr, and 30 W applied to the RF coil. After removal from the plasma chamber the TPE piece was brought into contact with the glass. The TPE‐glass chip was then left to cure in a 60 °C oven for 5 min. To enable pressure‐driven flow through the TPE‐glass hybrid devices, tubing connectors (Nanoport, WA) were attached to the chip using room‐temperature cured epoxy.

*Experimental Setup and Method*: During each experiment, the 2 µm red or 0.92 µm green fluorescent polystyrene microsphere suspensions (Thermo Scientific, MA) with specific concentrations (0.01, 0.1, and 1 v/v%) in deionized (DI) water were pumped into the microfluidic device at varying flow rates using a high‐pressure injection syringe pump (Harvard Apparatus, MA) to generate a stable and continuous flow. The inlet of the device was connected to a syringe by Tygon tubing. For the cell experiments, known concentrations (0.01 v/v% ≈ 2.5 × 10^7^ mL^−1^; 0.1 v/v% ≈ 2.5 × 10^8^ mL^−1^; and 1 v/v% ≈ 2.5 × 10^9^ mL^−1^) of cyanobacteria culture in the syringe were pumped into the device in the same manner as fluorescent microspheres. The suspending medium for the cyanobacteria in the inertial focusing experiments is a liquid BG‐11 medium.

*Cyanobacteria Source and Cultivation*: Cyanobacterium *Synechocystis* sp. PCC 6803 was grown in liquid BG‐11 medium.^46^ The strain was inoculated at an initial concentration of 10^6^ mL^−1^ and cultured in 250 mL Erlenmeyer flask with 50 mL culture medium in an INNOVA 44 Incubator Shaker (New Brunswick Scientific, Enfield, CT) at a speed of 225 rpm under 30 °C at an average light intensity of 100 ± 9 µmol m^−2^ s^−1^. These 50 mL suspensions were cultured to a final concentration between 2 × 10^8^ and 5 × 10^8^ mL^−1^. The suspension was then diluted or concentrated to achieve the desired density for the inertial focusing studies.

*Fluorescence Imaging*: TPE‐glass devices were mounted onto the stage of an inverted fluorescent microscope (AMG, Mill Creek, WA). Fluorescent streak images were obtained using a GFP light cube (excitation/emission: 470/510 nm) with exposure times of 200 ms. Recorded images were processed using ImageJ (http://rsb.info.nih.gov/ij/).

*High‐Speed Imaging*: The flow of the cyanoabcteria suspension was recorded at 2000 frames s^−1^ (495 µs interval) with a 5 µs shutter speed using a high‐speed camera (Fastcam SA3, Photron, USA) connected to an Olympus IX 71 Inverted optical Microscope (Olympus, Japan).

*Image Analysis and Measurement*: Image analysis was conducted using ImageJ (http://rsb.info.nih.gov/ij/). The concentrations of cyanobacteria were measured by a hemocytometer (hausser Scientific Partnership, Horsham, PA), and the suspension concentrations were then calculated from three different hemocytometry measurements.

## Conflict of Interest

The authors declare no conflict of interest.

## Supporting information

Supplementary
