# Supplementary material for: High‐Throughput Inertial Focusing of Micrometer‐ and Sub‐Micrometer‐Sized Particles Separation
Source: Adv Sci (Weinh). 2017 May 30;4(10):1700153. doi: 10.1002/advs.201700153 (PMC5644225; doi:10.1002/advs.201700153)
Supplement: Supplementary file 1 — Supplementary [file ADVS-4-na-s001.pdf]

Copyright WILEY-VCH Verlag GmbH & Co. KGaA, 69469 Weinheim, Germany, 2017.

ADVANCED SCIENCE Open Access

Supporting Information

for *Adv. Sci.,* DOI: 10.1002/advs.201700153

High-Throughput Inertial Focusing of Micrometer- and Sub-Micrometer-Sized Particles Separation

Lei Wang and David S. Dandy*

Supplementary Information

High-throughput inertial focusing of micron- and submicron-sized particles separation

Lei Wang,[a] and David S. Dandy

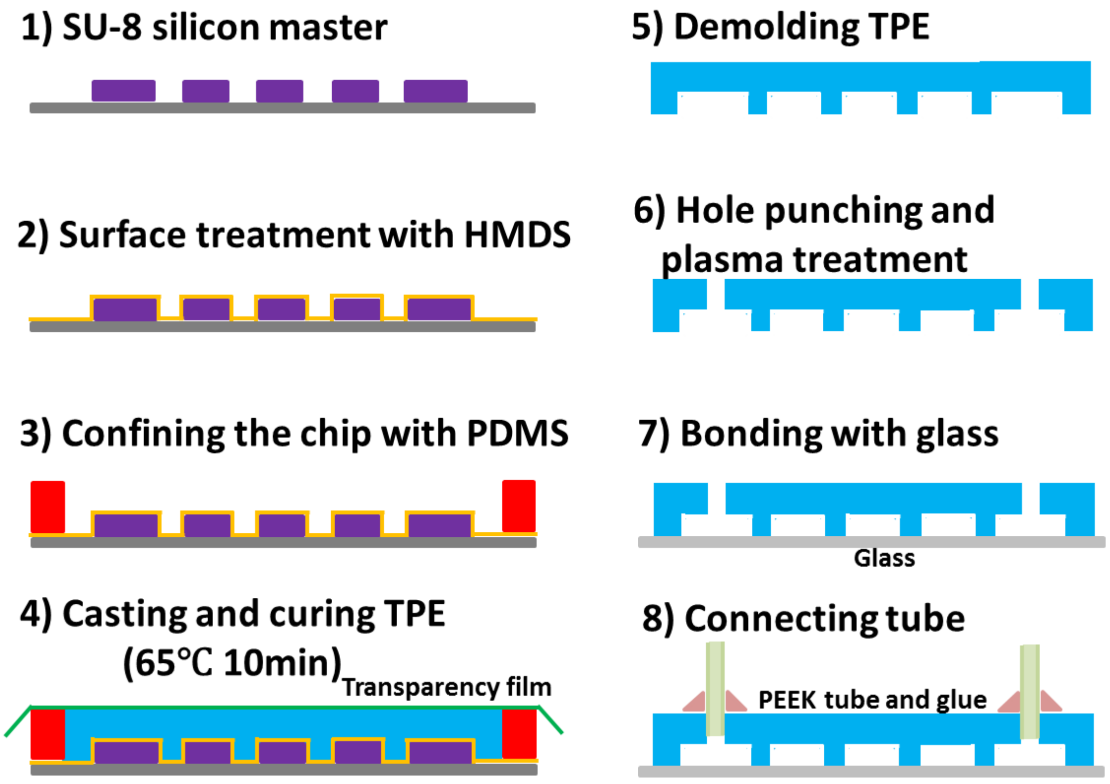

Supplementary Figure S1. Schematics illustrating the procedure to fabricate the thermoset polyester (TPE) inertial focusing microfluidic system.

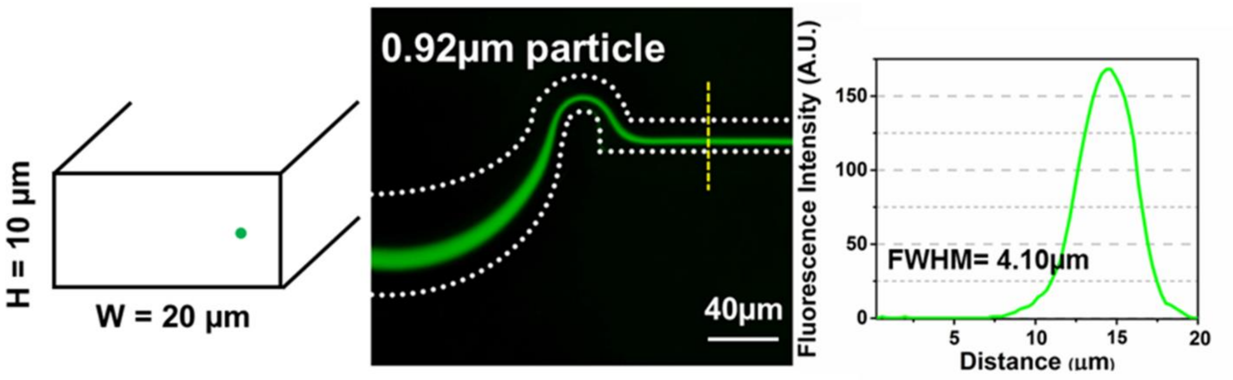

Supplementary Figure S2. Fluorescence image of 0.92 µm-sized green fluorescent particles in the final curve of the serpentine microchannel. The corresponding fluorescence intensity profile across the width of the straight channel region (w = 20 µm, h = 10 µm). The FWHM is calculated from a fitted Gaussian curve. The white dashed lines represent the microchannel boundaries.

To investigate the minimum length of serpentine channel required to focus 2 µm particles, additional serpentine channels, ranging in length from 1 mm to 4 mm, were designed and tested with a 0.01 v/v% suspension concentration. The resulting fluorescence images for these different microchannel lengths are shown in Supplementary Figure. S4 for a range of flow rates. The degree of focusing as a function of flow rate and channel length follows an interesting pattern. In the longest channel (4 mm), the 2 µm particles are focused at all flow rates 100 µL/min and higher, whereas in the 3 mm microchannel the particles are only focused at intermediate flow rates; and in the 2 mm microchannel focusing only occurs at a single flow rate. In the shortest channel, focusing isn't observed at any flow rate. Together, these results indicate that a minimum channel length (6 serpentine units, or 2 mm) and a minimum flow rate (100 µL/min) are both required to successfully focus 2 µm particles. An important advantage gained by obtaining a shorter focusing distance is decreased hydraulic resistance, and therefore, decreased pressure drop and power required to drive the flow.

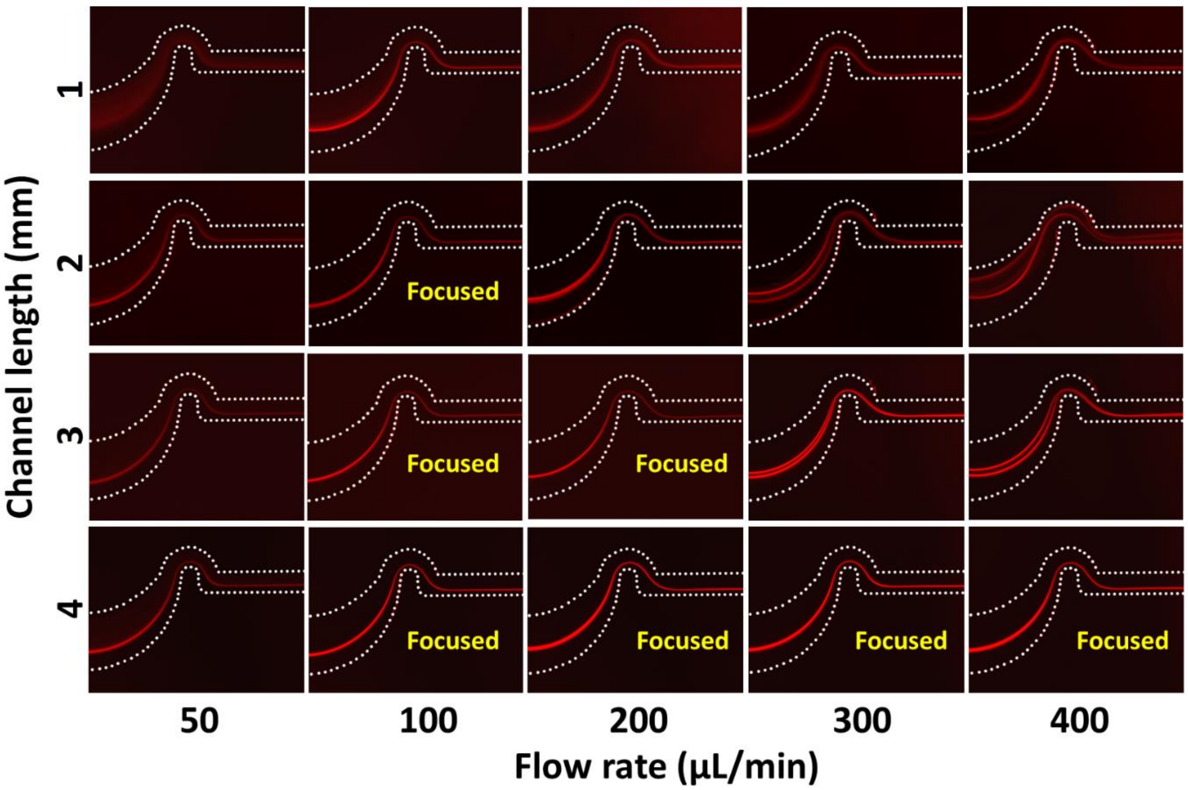

Supplementary Figure S3. The minimum serpentine microchannel length required for inertial focusing of micron-sized particles. Fluorescence images of 2 µm red fluorescent particles in the final curve of the serpentine microchannel for serpentine channel lengths ranging from 1 mm to 4 mm and flow rates ranging from 50 µL/min (Re = 55.4) to 400 µL/min (Re = 443). The white dashed lines represent the microchannel boundaries.

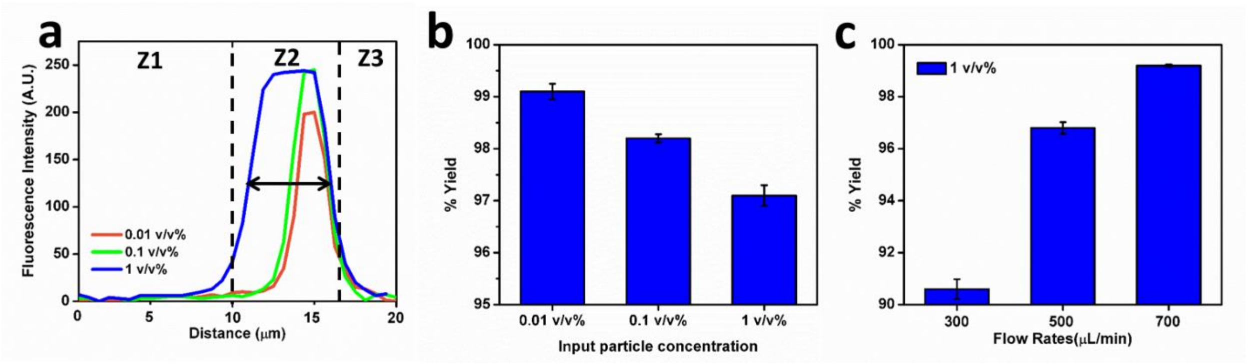

Supplementary Figure S4. The concentration profiles of the 2 µm particle suspensions for a range of inlet suspension densities. (a) The outlet design is based on the predicted locations of focused particle streams for separation into three zones. Z1 and Z3 are intended for the waste (particle-free) streams, and Z2 is designed for the concentrated product stream. (b) The yield percentage of 2 µm particles at a flow rate of 500 µL/min. (c) the yield percentage of 2 µm particles in the 1 v/v% suspension at three different flow rates. Error bars represent the standard deviation with a sample size of three.

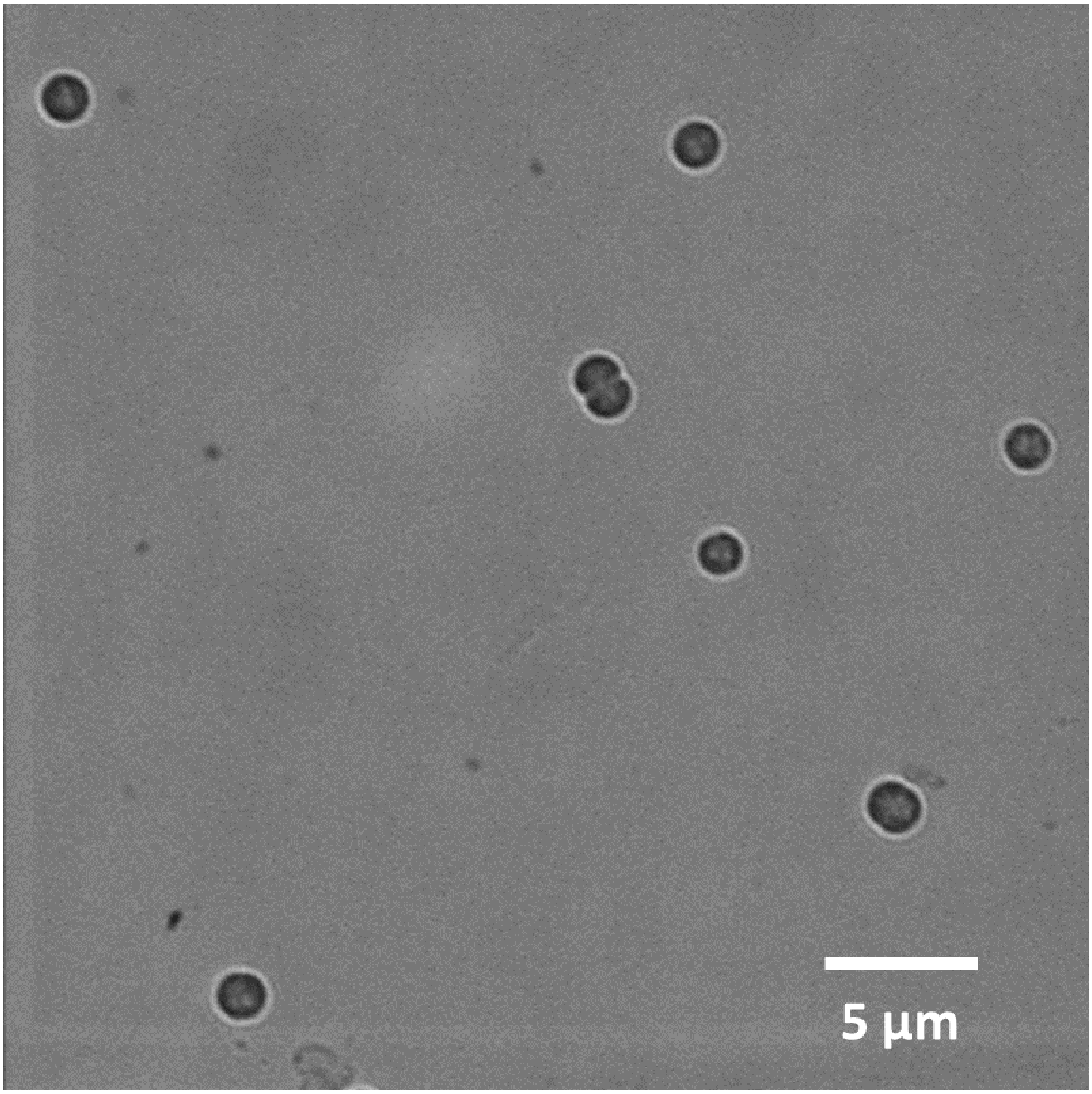

Supplementary Figure S5. An image of cultured cyanobacteria at 100× magnification. Although most of the cyanobacteria are spherical in shape under quiescent conditions, they will form a transient ellipsoidal shape while undergoing division.
